# Sequence-selective pulldown of recognition-encoded melamine oligomers using covalent capture on a solid support†

**DOI:** 10.1039/d4cc06018k

**Published:** 2024-12-06

**Authors:** Luis Escobar, Daniel Sun, Mohit Dhiman, Christopher A. Hunter

**Affiliations:** a Yusuf Hamied Department of Chemistry, University of Cambridge Lensfield Road Cambridge CB2 1EW UK herchelsmith.orgchem@ch.cam.ac.uk

## Abstract

The covalent capture of recognition-encoded melamine oligomers (REMO) with a target attached to a solid support was investigated. Sequence-selective pulldown of complementary oligomers was observed when the target was challenged with a randomised library of oligomers. The approach provides an affinity selection method for the discovery of functional REMO sequences.

## Abstract 2

Covalent capture on polymer beads provides an affinity selection method for the discovery of functional oligomers in mixed sequence libraries of synthetic polymers.

## Graphical abstract

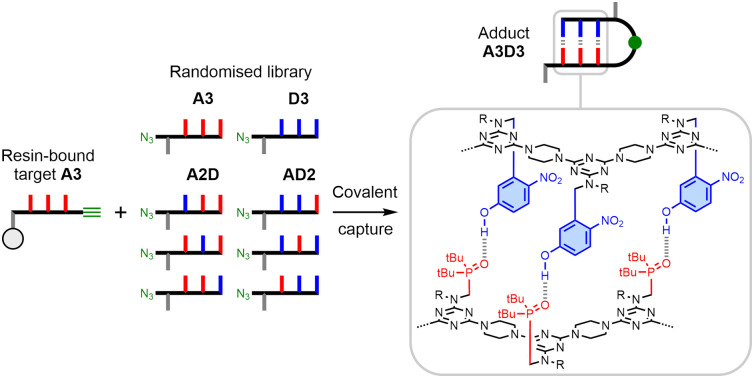

Affinity selection methods have become fundamentally important routinely-employed tools in biochemical research.^1,2^ Randomised libraries of biopolymers, such as nucleic acids or peptides, are used to identify sequences that exhibit high-binding affinities and selectivities for specific molecular targets.^3–6^ These targets are usually attached to a solid support either directly or *via* biotin–streptavidin linkages, which facilitates straightforward separation of free and bound components.^7–10^ As a result, affinity selection methods have enabled the rapid discovery of functional biopolymers, for example, molecular sensors and therapeutic agents.^11–14^ However, the use of synthetic randomised libraries in selection approaches is a relatively unexplored area.^15,16^

Synthetic polymers composed of different monomer units have the potential to recapitulate the sequence–function relationships found in biopolymers.^17–20^ We have developed one such class of polymer, recognition-encoded melamine oligomers (REMO), which have a uniform alternating 1,3,5-triazine-piperazine backbone and are equipped with phenol and phosphine oxide side-chains that define the sequence.^20^ REMO can be prepared in a straightforward manner by automated solid-phase synthesis from dichlorotriazine building blocks and piperazine using sequential nucleophilic aromatic substitution (S_N_Ar) reactions.^21,22^ Moreover, sequence-complementary REMO self-assemble into duplexes with high fidelity in non-polar solvents, such as dichloromethane (DCM), through H-bonding interactions between the 4-nitrophenol and phosphine oxide side-chains (Fig. 1).^22,23^ These characteristics make REMO attractive candidates for the implementation of affinity selection methods. Here, we report selective covalent capture of oligomers from REMO libraries based on H-bonding interactions with a target attached to a solid support.

**Fig. 1:**
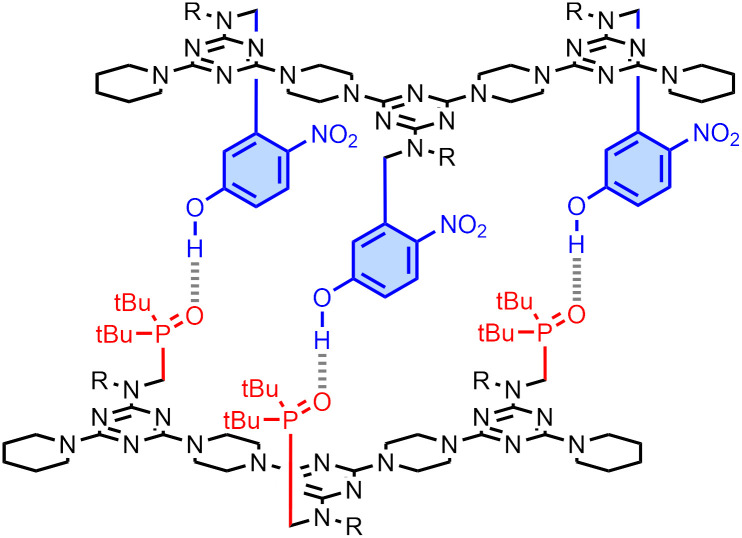
Structure of a H-bonded duplex assembled from two sequence-complementary REMO, pDDDp and pAAAp (the nomenclature used to define the oligomer sequence is D or A for H-bond donor or acceptor side-chains, and p, y or z for terminal piperidine, alkyne or azide units). R = alkyl groups to aid solubility in non-polar solvents.

The REMO 4-mers shown in Fig. 2a and b were prepared with an automated solid-phase synthesiser, as described previously (Schemes S1 and S3, ESI†).^21–26^ The first recognition unit in the chain was a phenol (denoted D′) in all cases, because this group was used to attach the growing oligomer to the resin in the synthesiser. The other positions had either 4-nitrophenol or phosphine oxide units (D and A, respectively). An AAA sequence was synthesised as the resin-bound target (Fig. 2a), and this oligomer was equipped with a terminal alkyne group for use in covalent capture experiments *via* copper-catalysed azide–alkyne cycloaddition (CuAAC) reactions.^27–29^ A randomised library of all possible REMO sequences, zD’XXXp (X = D or A), each equipped with a terminal azide group, was prepared by using a 3 : 2 mixture of 4-nitrophenol and phosphine oxide dichlorotriazine building blocks in each coupling step carried out on the synthesiser (Fig. 2b). The composition of this dichlorotriazine mixture compensated for the slight reactivity difference between the two building blocks (Scheme S2 and Fig. S2, S3, ESI†) and resulted in a statistical mixture of all possible sequences (*i.e.* eight different 4-mers in equal amounts). Two discrete oligomers equipped with a terminal azide group, zD′DDDp and zD′AAAp, were also synthesised for proof of principle covalent capture experiments (Fig. 2b).

**Fig. 2:**
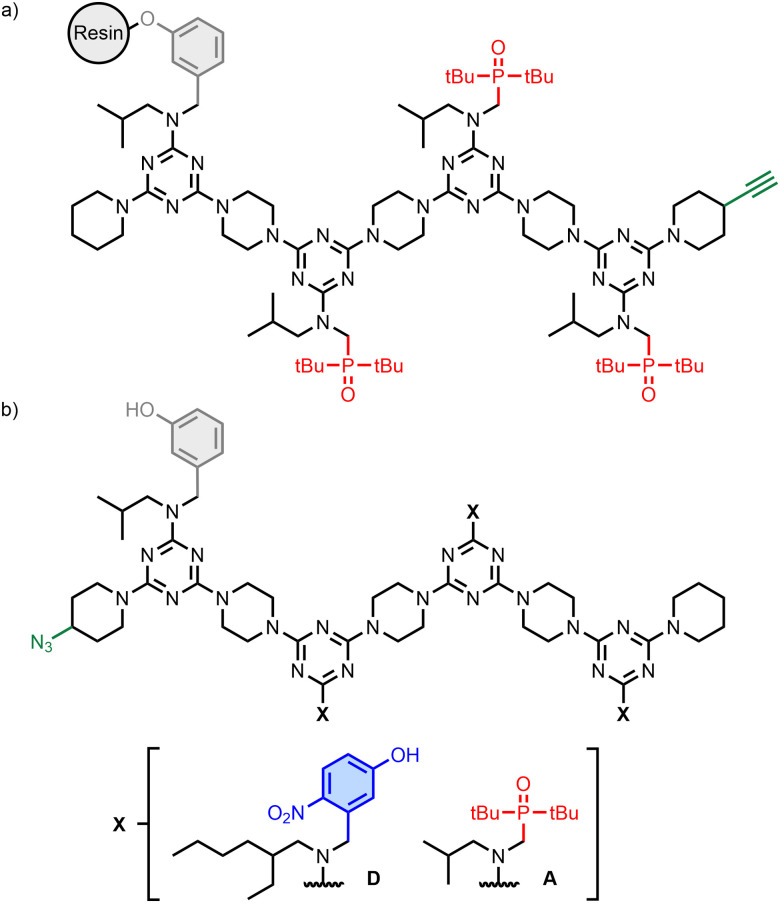
(a) Structure of the AAA target attached to the solid support. (b) Structures of the randomised REMO library, zD′XXXp, and discrete oligomers, zD′DDDp and zD′AAAp.

In order to establish the methodology for the covalent capture experiment using the CuAAC reaction, a 1 : 1 mixture of zD′DDDp and zD′AAAp was reacted with the resin-bound AAA target. The association constant for the duplex shown in Fig. 1, which forms three 4-nitrophenol⋅phosphine oxide H-bonds is 4.7 × 10^6^ M^−1^ in DCM, so at concentrations greater than 10 μM, zD′DDDp should be fully bound to either AAA on the resin or zD′AAAp in solution.^30^ In contrast, zD′AAAp can only interact with AAA on the resin *via* one H-bond with the terminal phenol, which is a much worse H-bond donor than the 4-nitrophenol groups, so at μM concentrations, there will be no interaction between this oligomer and the target.

The resin-bound AAA target (*ca.* 0.5 μmol) was suspended in DCM, followed by the addition of zD′DDDp (1 μmol, 0.2 mM), Cu(MeCN)_4_PF_6_ (1 μmol, 0.2 mM) and tris(benzyltriazolylmethyl)amine (TBTA) (1 μmol, 0.2 mM). After shaking at room temperature (r.t.) for 2 days, the resin was filtered off and washed with DCM and dimethylformamide (DMF). The covalently-attached reaction products were cleaved from the resin with trifluoroacetic acid (TFA), DCM and triisopropylsilane (TIS) in a 90 : 5 : 5 ratio at r.t. for 2 h, and the resulting mixture was analysed by ultra-performance liquid chromatography – mass spectrometry (UPLC-MS). The UPLC trace revealed that the starting alkyne had been quantitatively converted to the A3D3 adduct (Fig. S20a, b and Table S5, ESI†). When the same experiment was carried out using zD′AAAp, the starting alkyne was converted to the A6 adduct (Figure S22a, b and Table S6, ESI†). Therefore, a competition experiment was carried out to test whether the product distribution of the CuAAC reaction could be influenced by H-bonding interactions with the reactants (Fig. 3a).

**Fig. 3:**
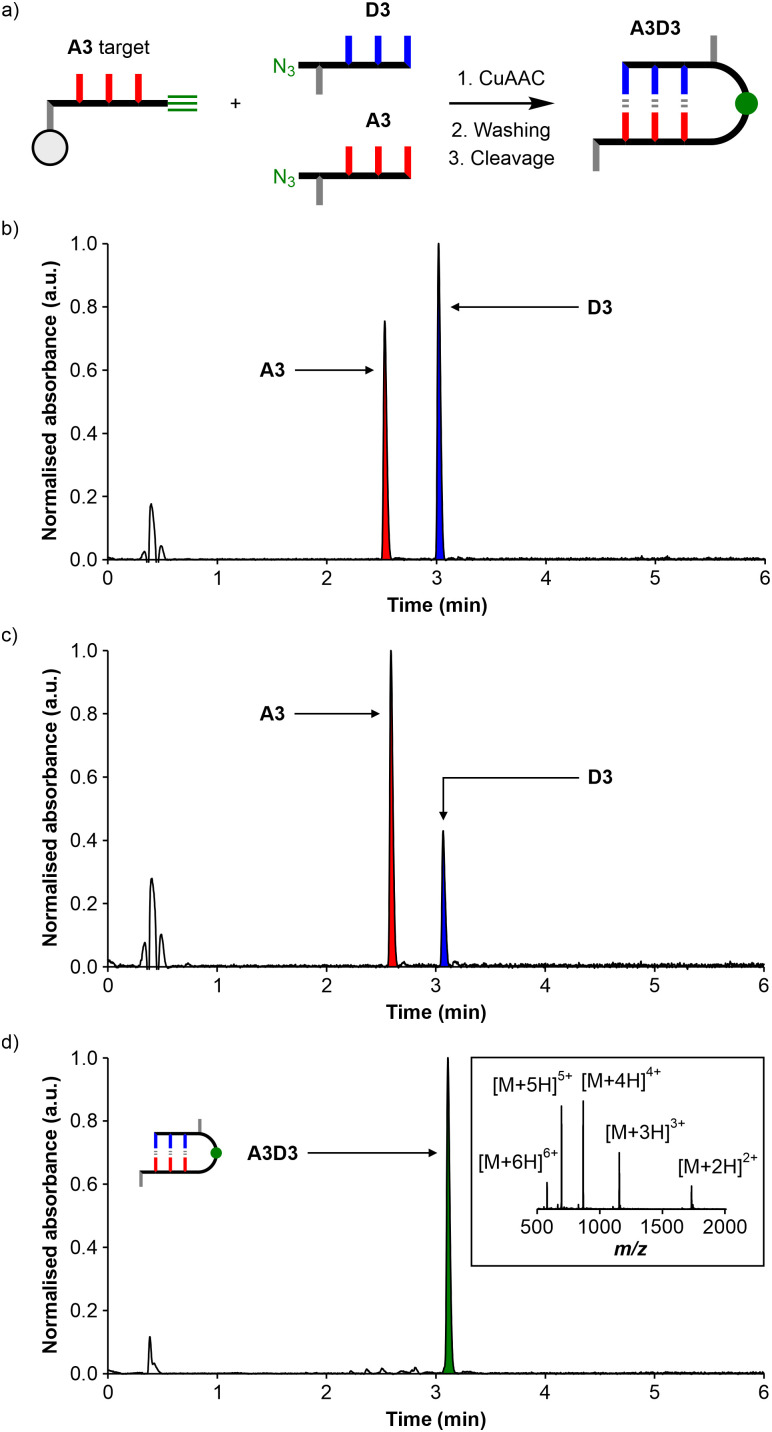
(a) Schematic representation of the covalent capture experiment using a mixture of zD′DDDp and zD′AAAp in solution and a resin-bound AAA target. UPLC traces: (b) 1 : 1 mixture of zD′DDDp and zD′AAAp (peaks with slightly different absorption due to oligomers composition); (c) solution phase collected after the CuAAC reaction by filtration and washing of the reacted resin and (d) crude product mixture obtained after cleavage from the resin (inset shows the corresponding mass spectrum of the A3D3 peak).

A 1 : 1 mixture of zD′DDDp (1 μmol, 0.2 mM) and zD′AAAp (1 μmol, 0.2 mM) was reacted with the resin-bound AAA target (*ca.* 0.5 μmol) in DCM using the protocol described above. Fig. 3b and c show the UPLC traces of the solution phase before and after the CuAAC reaction, respectively. The large decrease in the intensity of the peak due to the D3 oligomer suggests that this compound reacted selectively with the resin-bound target. The UPLC trace of the product mixture obtained after cleavage from the resin supported this observation, because the only product obtained was the A3D3 adduct (Fig. 3d). These observations confirm that the CuAAC covalent capture experiment can be used to effectively probe the selectivity of non-covalent binding interactions between a mixture of oligomers in solution and a target attached to the resin. Although both the A3 and D3 oligomers react with the target, in a competition experiment, only the oligomer that forms a high affinity complex with the target was captured.

The covalent capture experiment was then repeated using the randomised library, zD′XXXp, to assess the sequence-selectivity of duplex formation in a more complex mixture. Fig. 4a shows the eight sequences present in the library, and the UPLC trace in Fig. 4b suggests that they are present as a statistical mixture. We have not been able to separate isomeric sequences by UPLC, but sequences with different compositions are well-resolved, and the A3, A2D, AD2 and D3 compositions are present in the expected 1 : 3 : 3 : 1 ratio. The library (8 μmol, 1.6 mM) was reacted with the resin-bound AAA target (*ca.* 0.5 μmol) in DCM using the protocol described above, and resulting UPLC traces are shown in Fig. 4c and d. The solution phase filtered off after the CuAAC reaction showed a decrease in the proportion of D rich sequences (Fig. 4c), and the product mixture obtained after cleavage from the resin confirmed this observation (Fig. 4d, Fig. S28 and Table S9, ESI†). Three covalent adducts were detected: A3D3 (approximately 75%), A4D2 (approximately 25%) and A5D (trace). When the concentration of the randomised library used in the covalent capture experiment was diluted by a factor of 10, the selectivity for the fully sequence-complementary oligomer increased further to approximately 80% for A3D3 (approximately 20% for A4D2) (Fig. S27, ESI†).

**Fig. 4:**
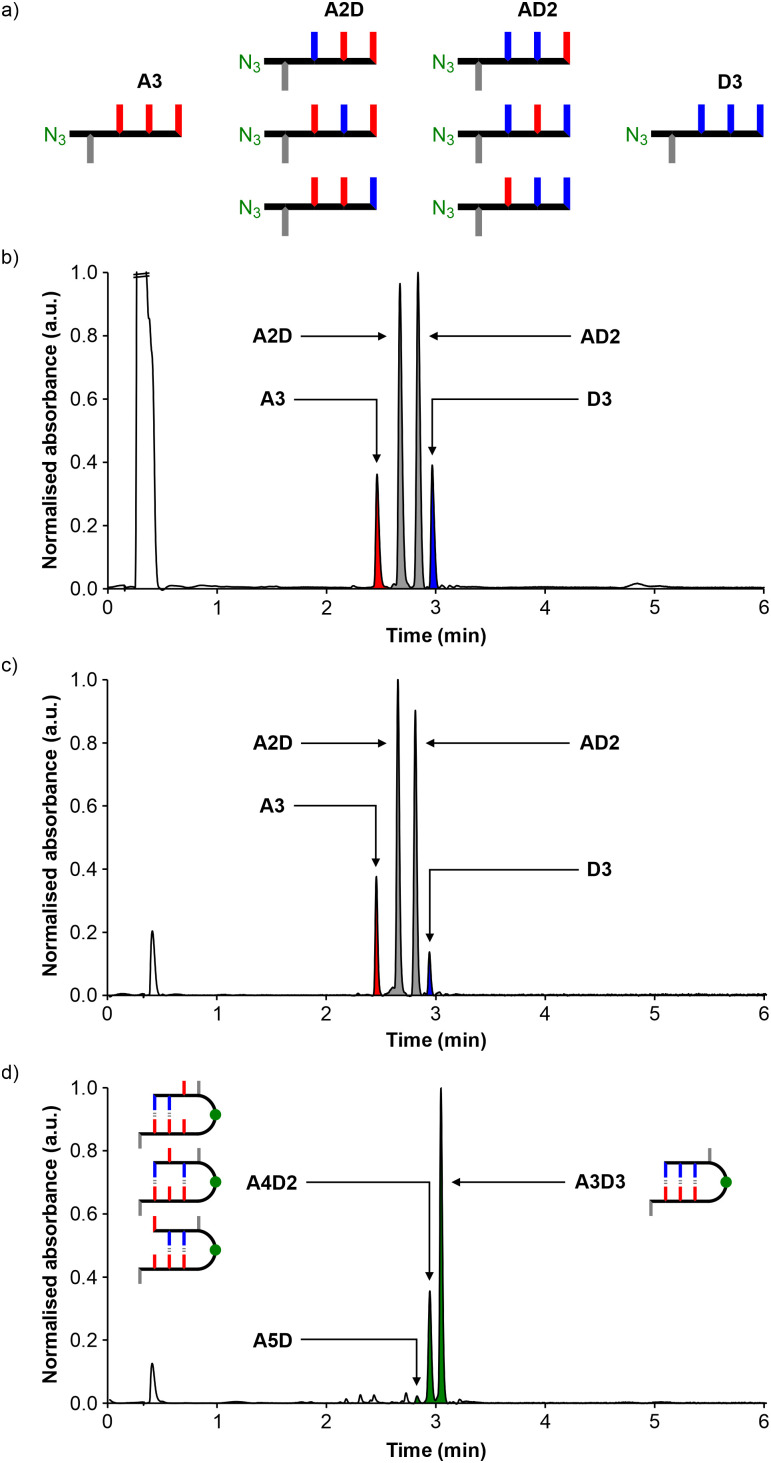
(a) Schematic representation of the randomised library of REMO 4-mers, zD′XXXp. UPLC traces of the covalent capture experiment with a resin-bound AAA target: (b) REMO library; (c) solution phase collected after the CuAAC reaction by filtration and washing of the reacted resin and (d) crude product mixture obtained after cleavage from the resin.

The high yield of the sequence-complementary A3D3 adduct obtained in the covalent capture experiment suggests that the selectivity is indeed governed by sequence-selective duplex formation. However, the amount of single mismatch product, A4D2, is also significant. One reason for this is that there are three different AD2 oligomers that compete with D3 for binding to the target. In other words, the 4 : 1 selectivity for A3D3 over A4D2 reflects a 12 : 1 difference in binding affinity for D3 relative to AD2, if all the oligomers are present in equal amounts. Since the A2D and AD2 oligomers are present in equal amounts, the yield of the double mismatch product, A5D, should be 12-fold lower than A4D2, which explains why the A5D adduct is barely detectable. A more quantitative interpretation of these results is compromised by the fact that the solution phase oligomers may form intramolecular H-bonds or bind with one another, and these species compete with binding to the target. Nevertheless, it is clear that the major product is the highest affinity binding partner present in the library.

In summary, we have demonstrated selective covalent capture of oligomers from mixed sequence REMO libraries. A CuAAC reaction with a resin-bound target equipped with an alkyne was used to pulldown the highest affinity binding partner from solution, and UPLC-MS was used to identify the sequence. This result augurs well for the application of affinity selection methods to explore the functional properties of REMO.

We thank the European Research Council (ERC-2020-AdG-101018984-InfoMols) for financial support. We also thank Cecilia J. Anderson and Laura Beale for synthetic assistance.

## Conflicts of interest

There are no conflicts to declare.

## Supplementary Material

CC-061-D4CC06018K-s001

## Data Availability

The data supporting this article have been included as part of the ESI.†
