# Supplementary material for: Sequence-selective pulldown of recognition-encoded melamine oligomers using covalent capture on a solid support
Source: Chem Commun (Camb). 2024 Dec 6;61(3):504–7. doi: 10.1039/d4cc06018k (PMC11622007; doi:10.1039/d4cc06018k)
Supplement: CC-061-D4CC06018K-s001 [file CC-061-D4CC06018K-s001.pdf]

Supplementary Information (SI) for ChemComm.
This journal is © The Royal Society of Chemistry 2024

Sequence-selective pulldown of recognition-encoded melamine oligomers using covalent capture on a solid support

Luis Escobar,[†,a] Daniel Sun[†,a] Mohit Dhiman [a] and Christopher A. Hunter*[,a]

[a] Yusuf Hamied Department of Chemistry, University of Cambridge, Lensfield Road, Cambridge CB2 1EW, United Kingdom

[†] These authors contributed equally.

E-mail: herchelsmith.orgchem@ch.cam.ac.uk

Supporting Information

Table of Contents

1. General information and instruments S2
2. Synthesis of building blocks S2
3. Functionalization of the resin S2
4. Relative reactivities of building blocks S3
5. Synthesis and characterization of oligomers S4
   5.1 Alkyne-oligomer with three phosphine oxide units S5
   5.2 Azide-oligomer with three 4-nitrophenol units S6
   5.3 Azide-oligomer with three phosphine oxide units S8
   5.4 Randomized library of azide-oligomers S9
6. Covalent capture of oligomers S11
   6.1 Reaction with the azide-oligomer containing three 4-nitrophenol units S12
   6.2 Reaction with the azide-oligomer containing three phosphine oxide units S13
   6.3 Reaction with a mixture of two azide-oligomers S14
   6.4 Reactions with a randomized library of azide-oligomers S15
7. References S18

1. General information and instruments

Reagents were obtained from commercial suppliers and used without further purification unless otherwise stated. All solvents were commercially obtained and used without further purification. Dry solvents were taken from a Solvent Purification System (SPS) PS-MD-5. Routine ^{1}H NMR and $^{13}C\{^{1}H\}$ NMR spectra were recorded on a Bruker 400 MHz Avance III HD Smart Probe (400 MHz for ^{1}H NMR and 100 MHz for ^{13}C NMR), Bruker 400 MHz Neo Prodigy with cryoprobe (400 MHz for ^{1}H NMR and 100 MHz for ^{13}C NMR) and Bruker 500 MHz Avance III Smart Probe (500 MHz for ^{1}H NMR and 125 MHz for ^{13}C NMR) spectrometers. Deuterated solvents used are indicated in the characterization and chemical shifts are given in ppm. Residual solvent peaks were used as reference.[1] All NMR *J* values are given in Hz. Microwave reactions were performed on a Biotage Initiator+ apparatus. UV-Vis spectra were recorded on an Agilent Cary 60 UV-Vis spectrophotometer. Flash column chromatography was performed on a CombiFlash Rf+UV-Vis and a CombiFlash Rf+Lumen UV-Vis instruments using prepacked cartridges of silica gel. Reaction progress was monitored by TLC analysis on silica gel 60 F254 and using a Waters Acquity H-class UPLC (λ = 254 nm) coupled with a mass spectrometer and equipped with a Protein BEH C4 300 Å column (2.1 × 50 mm, 1.7 µm). Reverse-phase chromatography was performed on an analytical HPLC Agilent HP1100 and a preparative HPLC Agilent HP1100 (λ = 254 nm) equipped with a XBridge BEH C8 (4.6 × 75 mm, 2.5 µm) and a XBridge Prep C8 OBD (19 × 250 mm, 5 µm) columns, respectively. Melamine oligomers were prepared using a CEM Liberty Blue automated microwave peptide synthesizer and TentaGel S Wang resin (90 µm, *ca.* 0.24 mmol·g^{-1}).

2. Synthesis of building blocks

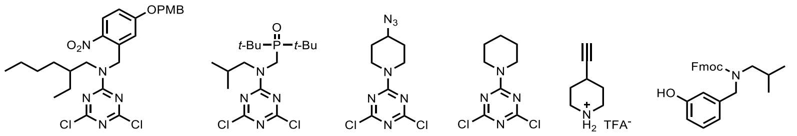

S1 S2 S3 S4 S5 S6

Figure S1. Line-drawing structures of building blocks **S1**-**S6**.

Compounds **S1**-**S6** were synthesized following procedures previously reported in the literature.[2,3,4,5,6,7,8]

3. Functionalization of the resin

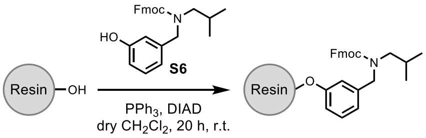

Scheme S1. Synthesis of functionalized resin.

Functionalized resin:[6,7] TentaGel S Wang resin (3.00 g) was swollen in dry CH_2Cl_2 (21 mL) for 30 min. Compound **S6** (0.65 g, 1.62 mmol) and PPh_3 (0.42 g, 1.62 mmol) were dissolved in dry CH_2Cl_2 (5.7 mL) and added to the resin suspension. DIAD (0.32 mL, 1.62 mmol) was dissolved in dry CH_2Cl_2 (1.5 mL) and added to the resin suspension. The reaction was stirred at r.t. for 20 h under nitrogen atmosphere. After that, the resin was filtered, washed with CH_2Cl_2 (4 × 25 mL) and dried under high vacuum. The loading of the functionalized resin was quantified by treating a sample (24 mg) with a solution of DBU in DMF (2 vol. %, 2.0 mL) at r.t. for 30 min. The solution mixture was diluted with MeCN (5-fold) and then further diluted with the same solvent (13.5-fold). The absorbance of the DBU-fulvene adduct (λ = 304 nm, ε = 9254 M^{-1}·cm^{-1}) was measured to estimate the resin loading (*ca.* 70 µmol·g^{-1}).

4. Relative reactivities of building blocks

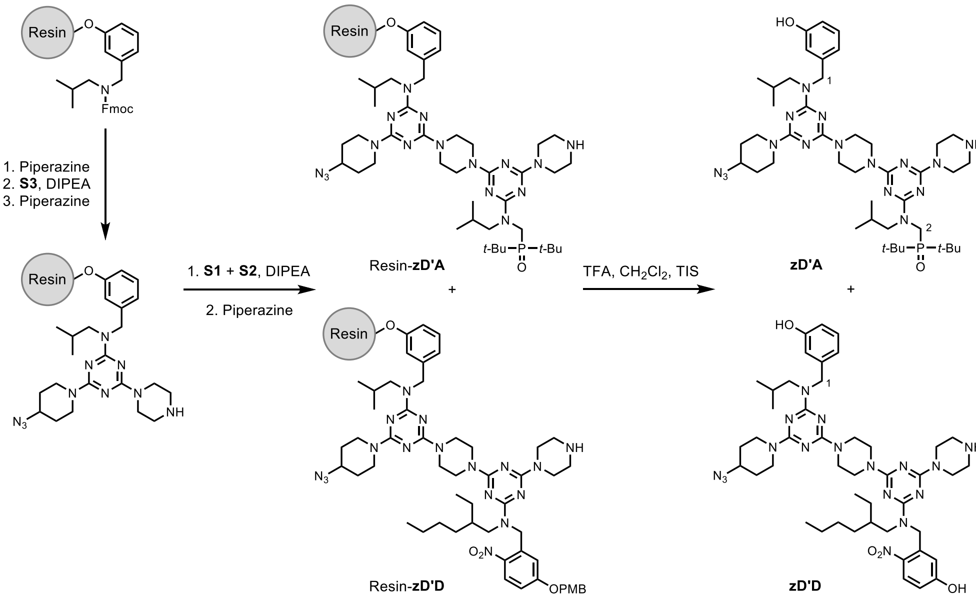

Scheme S2. Simultaneous incorporation of a mixture of building blocks **S1** and **S2** into the functionalized resin.

Relative reactivites: Simultaneous incorporation of building blocks **S1** and **S2** (3:2 molar ratio, 0.125 M) into the functionalized resin was performed following the general procedure described in Section 5. After that, the synthesized oligomers were cleaved from the resin with 90:5:5 TFA/CH_2Cl_2/TIS at r.t. for 2 h. The obtained solution mixture was concentrated and redissolved in DMSO-d_6 with 1,3,5-trimethoxybenzene as the internal standard (I.S.). Finally, a ^{1}H NMR spectrum of the mixture was acquired at 373 K (Figure S2 and Figure S3). Integration of selected proton signals (1 and 2, Scheme S2) indicated that the oligomers **zD'A** and **zD'D** were formed in equimolar amounts. Thus, **S1** and **S2** were incorporated into the functionalized resin in a 1:1 molar ratio when using an initial solution mixture of building blocks in a 3:2 molar ratio.

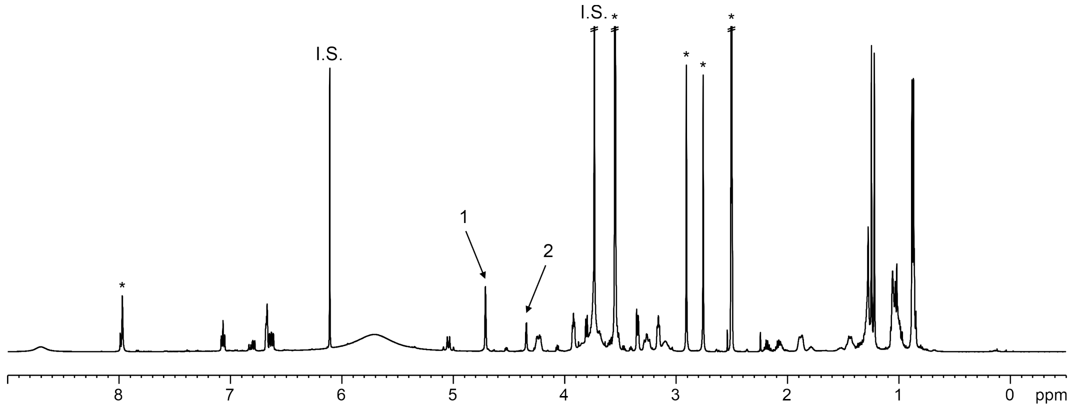

Figure S2. ^{1}H NMR (500 MHz, DMSO-d_6, 373 K) spectrum of the solution mixture containing **zD'A** and **zD'D**. See Scheme S2 for proton assignments. I.S. = 1,3,5-trimethoxybenzene. *Residual solvent peaks.

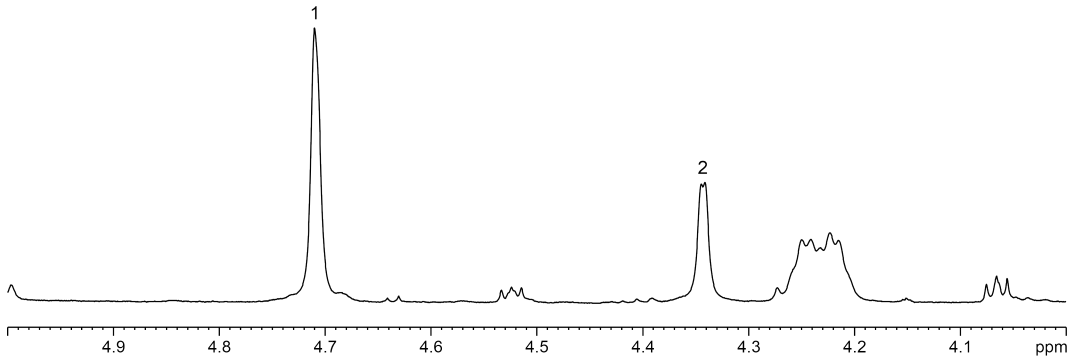

Figure S3. Selected region of the ^{1}H NMR (500 MHz, DMSO-d_6, 373 K) spectrum of the solution mixture containing **zD'A** and **zD'D**. See Scheme S2 for proton assignments.

5. Synthesis and characterization of oligomers

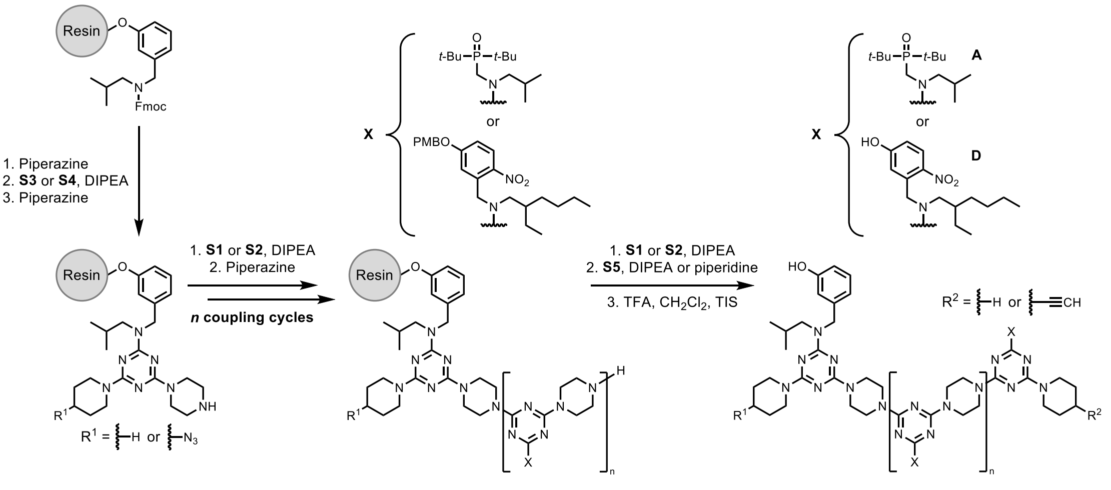

Scheme S3. Automated solid-phase synthesis of melamine oligomers.

General procedure:[6,7] Melamine oligomers were prepared using a CEM Liberty Blue automated microwave peptide synthesizer. Separate solutions of piperazine (*ca.* 0.7 M), DIPEA (0.5 M) and **S1**-**S4** (0.125 M) were prepared in DMF for peptide synthesis.

Fmoc-deprotection reaction: The functionalized resin (*ca.* 400 mg) was treated with piperazine in DMF (7 mL, *ca.* 0.7 M) at 18ºC for 10 min (× 2). The solution was drained, and the resin was washed with DMF (4 × 5 mL).

Coupling cycle: First, the resin was treated with **S1**-**S4** (0.1 M) and DIPEA (0.1 M) in DMF (5 mL) at 90ºC for 10-15 min. The solution was drained, and the resin was washed with DMF (4 × 5 mL). Second, the resin was treated with piperazine in DMF (5 mL, *ca.* 0.7 M) at 90ºC for 10-15 min. The solution was drained, and the resin was washed with DMF (4 × 5 mL).

Coupling of **S5** or piperidine (microwave reaction on a Biotage Initiator+ apparatus): The resin was treated with either a mixture of **S5** (0.1 M) and DIPEA (1 M), or piperidine (0.1 M), in DMF (5 mL) at 100ºC for 30 min. The solution was drained, and the resin was washed with DMF (4 × 5 mL) and CH_2Cl_2 (4 × 5 mL).

Cleavage of oligomer from the resin and deprotection of 4-nitrophenol units: The resin was treated with 90:5:5 TFA/CH_2Cl_2/TIS (5 mL) at r.t. for 2 h. After that, the crude was filtered, and the resin was washed with CH_2Cl_2 (4 × 5 mL). The crude was concentrated with a nitrogen flow and dried under high vacuum.

Purification of oligomer: The crude was redissolved in DMSO or THF/DMSO (1-2 mL), prior to analysis and purification by reverse-phase HPLC using a C8 column (see Figure footnotes for details).

5.1 Alkyne-oligomer with three phosphine oxide units

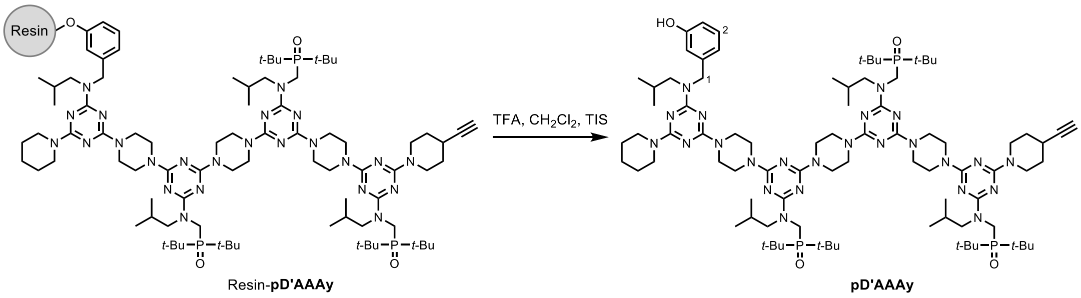

Scheme S4. Cleavage of oligomer **pD’AAAy** from the resin.

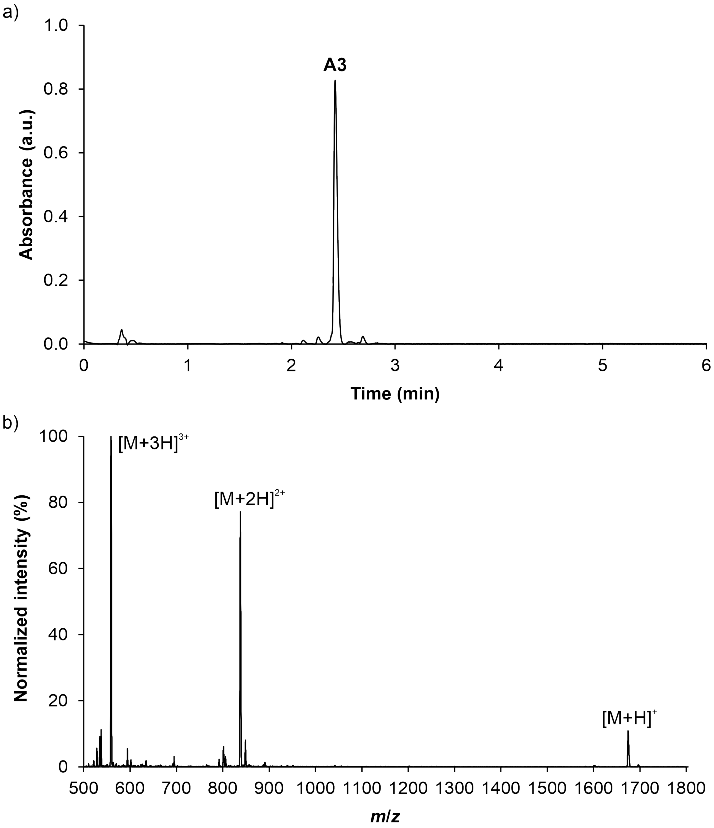

Figure S4. a) UPLC chromatogram of oligomer **pD’AAAy** after cleavage from the resin. Method (water/THF, containing 0.1% formic acid): 70:30 → 0:100 in 4 min and 0:100 for 2 min; flow rate = 0.4 mL·min^{-1}. b) Mass spectrum of **pD’AAAy**.

Table S1. Calculated and found mass (*m*/*z*) of the ions of **pD’AAAy**.

Formula	Calcd. *m*/*z*	Found *m*/*z*
$[M+H]^+$	1674.13	1674.63
$[M+2H]^{2+}$	837.57	837.97
$[M+3H]^{3+}$	558.72	558.95

Amount of oligomer attached to the resin: Resin-**pD'AAAy** (32 mg) was treated with 90:5:5 TFA/CH_2Cl_2/TIS (1 mL) at r.t. for 2 h. The obtained solution mixture was concentrated and redissolved in DMSO-d_6 with 1,3,5-trimethoxybenzene as the internal standard (I.S.). Finally, a ^{1}H NMR spectrum of the mixture was acquired at 373 K (Figure S5 and Figure S6). Based on the integral values of selected proton signals (1 and 2, Scheme S4) and those of the internal standard, we estimated a maximum amount of *ca.* 60 μmol·g^{-1} for the oligomer **pD'AAAy** attached to the resin.

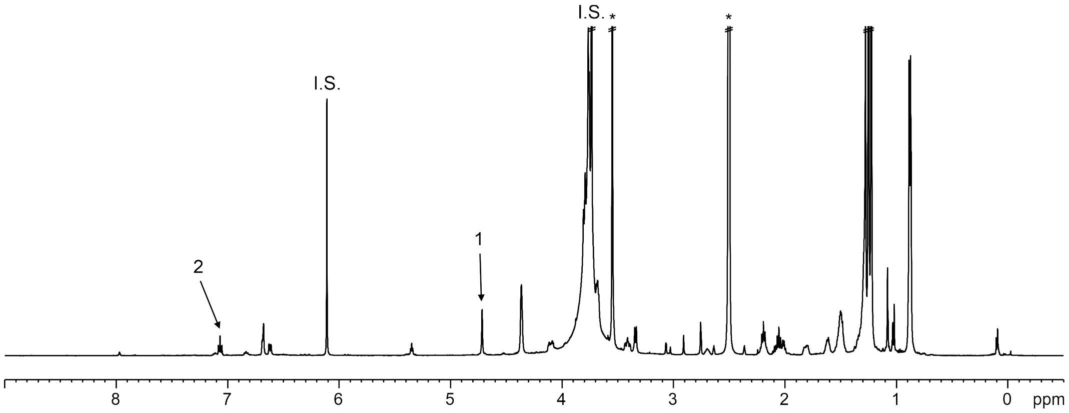

Figure S5. ^{1}H NMR (500 MHz, DMSO-d_6, 373 K) spectrum of oligomer **pD'AAAy** after cleavage from the resin. See Scheme S4 for proton assignment. I.S. = 1,3,5-trimethoxybenzene. *Residual solvent peaks.

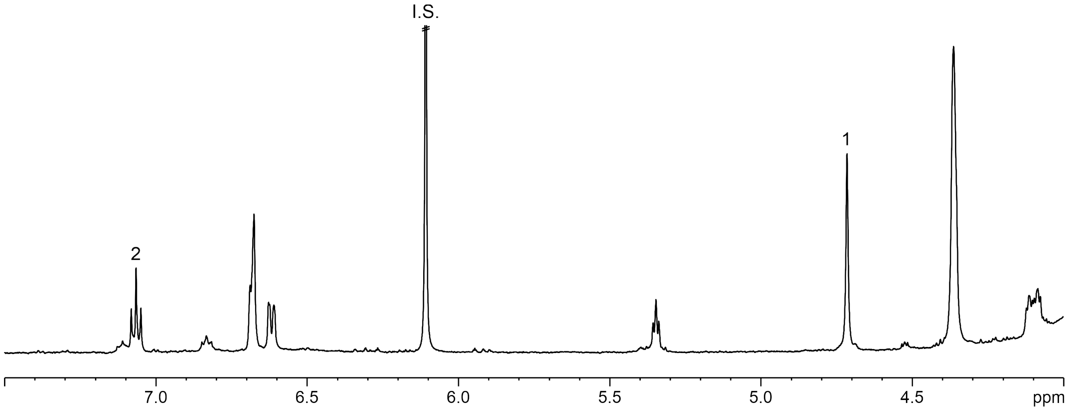

Figure S6. Selected region of the ^{1}H NMR (500 MHz, DMSO-d_6, 373 K) spectrum of oligomer **pD'AAAy** after cleavage from the resin. See Scheme S4 for proton assignment. I.S. = 1,3,5-trimethoxybenzene.

5.2 Azide-oligomer with three 4-nitrophenol units

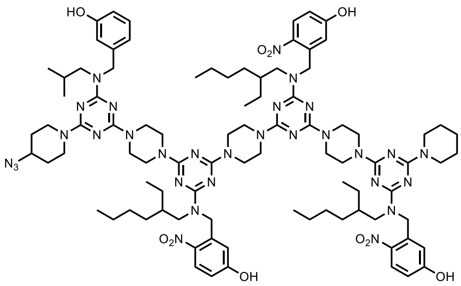

Figure S7. Line-drawing structure of oligomer **zD'DDDp**.

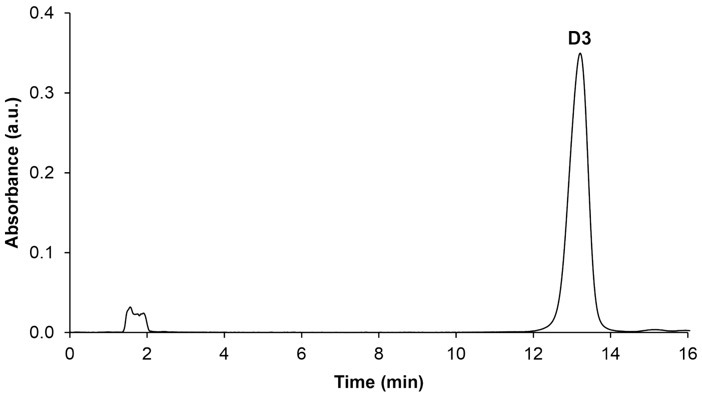

Figure S8. HPLC chromatogram of isolated oligomer **zD'DDDp**. Method ([95:5 water/MeCN]/THF): 35:65; flow rate = 0.5 mL·min^{-1}.

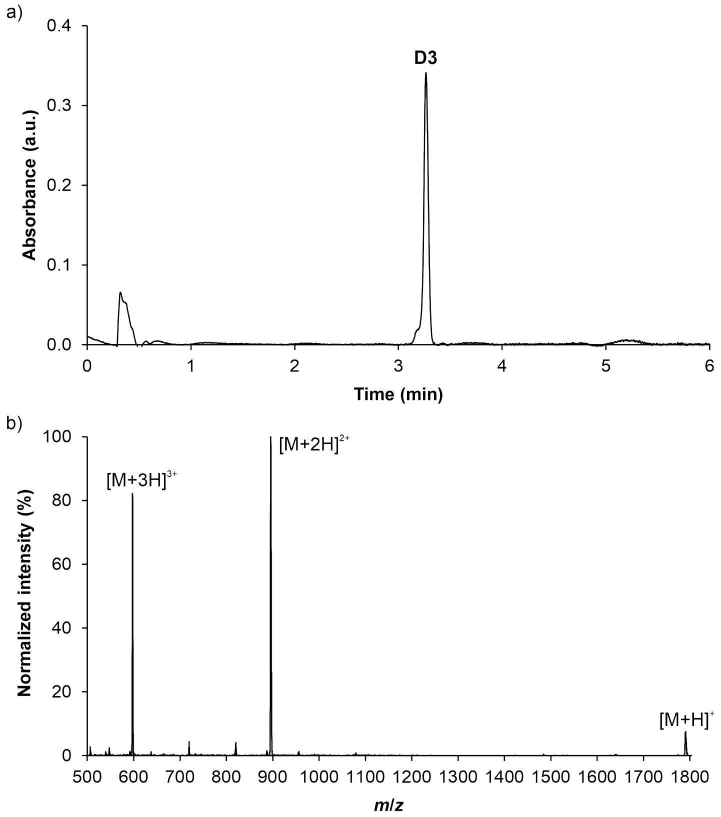

Figure S9. a) UPLC chromatogram of oligomer **zD'DDDp** after purification. Method (water/THF, containing 0.1% formic acid): 70:30 → 0:100 in 4 min and 0:100 for 2 min; flow rate = 0.4 mL·min^{-1}. b) Mass spectrum of **zD'DDDp**.

Table S2. Calculated and found mass (*m*/*z*) of the ions of **zD'DDDp**.

Formula	Calcd. *m*/*z*	Found *m*/*z*
$[M+H]^+$	1790.05	1791.15
$[M+2H]^{2+}$	895.53	895.91
$[M+3H]^{3+}$	597.35	597.64

5.3 Azide-oligomer with three phosphine oxide units

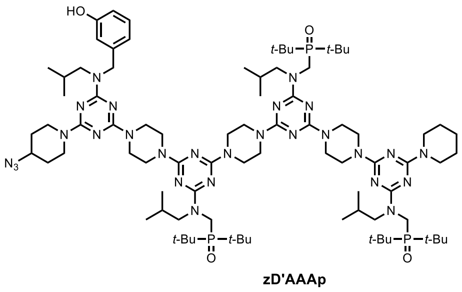

Figure S10. Line-drawing structure of oligomer **zD'AAAp**.

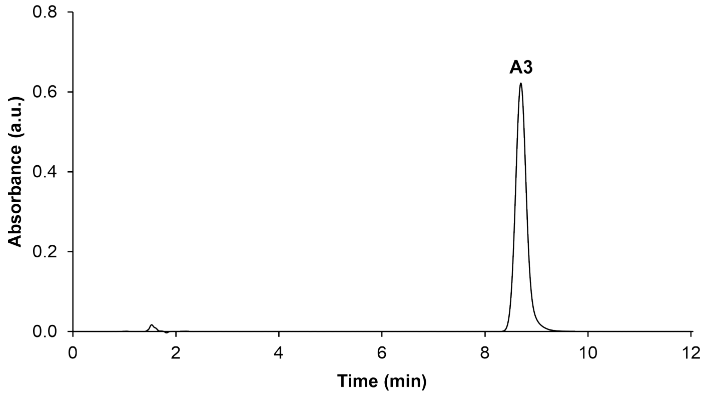

Figure S11. HPLC chromatogram of isolated oligomer **zD'AAAp**. Method ([95:5 water/MeCN]/THF): 44:56; flow rate = 0.5 mL·min^{-1}.

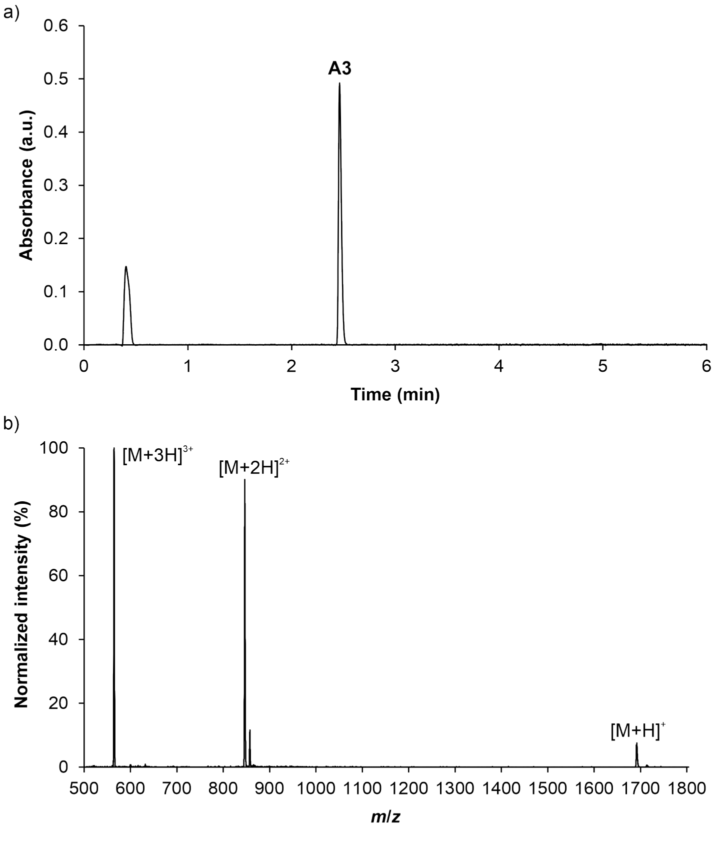

Figure S12. a) UPLC chromatogram of oligomer **zD'AAAp** after purification. Method (water/THF, containing 0.1% formic acid): 70:30 → 0:100 in 4 min and 0:100 for 2 min; flow rate = 0.4 mL·min^{-1}. b) Mass spectrum of **zD'AAAp**.

Table S3. Calculated and found mass (*m*/*z*) of the ions of **zD'AAAp**.

Formula	Calcd. *m*/*z*	Found *m*/*z*
$[M+H]^{+}$	1691.13	1692.38
$[M+2H]^{2+}$	846.07	846.47
$[M+3H]^{3+}$	564.38	564.57

5.4 Randomized library of azide-oligomers

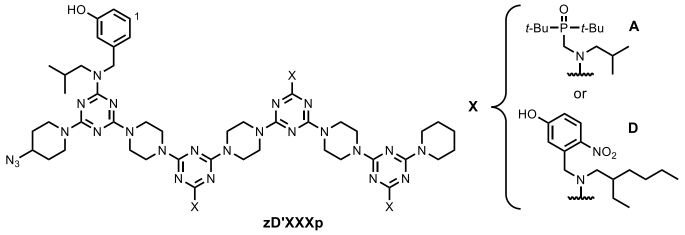

Figure S13. Randomized library of oligomers **zD'XXXp**. The incorporation of building blocks **S1** and **S2** in a 1:1 molar ratio led to the formation of a library composed of 8 oligomers: **A3**, 3 × **A2D**, 3 × **AD2** and **D3**.

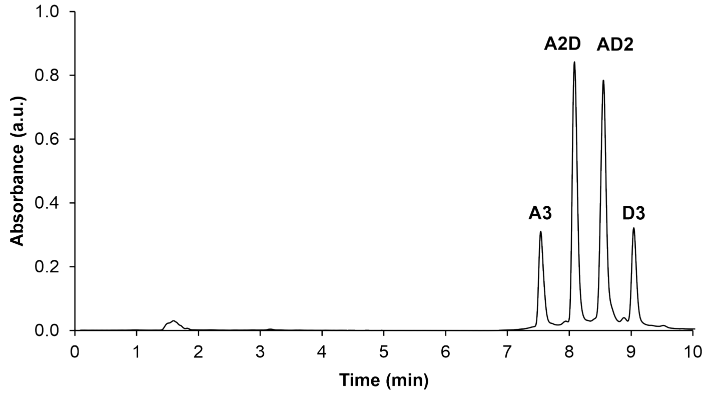

Figure S14. HPLC chromatogram of the library of oligomers **zD'XXXp** after purification. Method ([95:5 water/MeCN]/THF): 45:55 for 4 min; 45:55 → 20:80 in 1 min and 20:80 for 5 min; flow rate = 0.5 mL·min^{-1}.

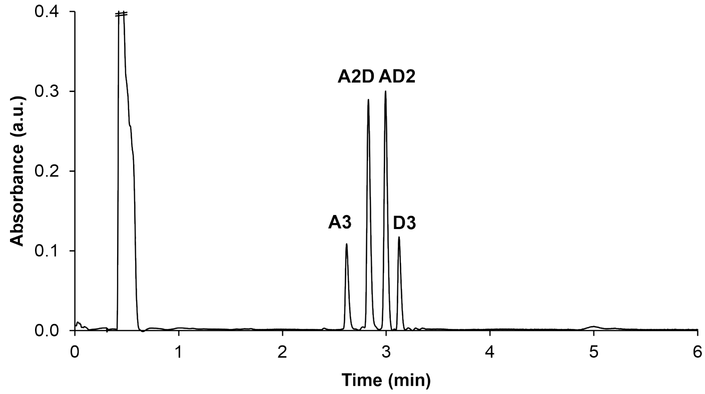

Figure S15. UPLC chromatogram of the library of oligomers **zD'XXXp** after purification. Method (water/THF, containing 0.1% formic acid): 70:30 → 0:100 in 4 min and 0:100 for 2 min; flow rate = 0.4 mL·min^{-1}.

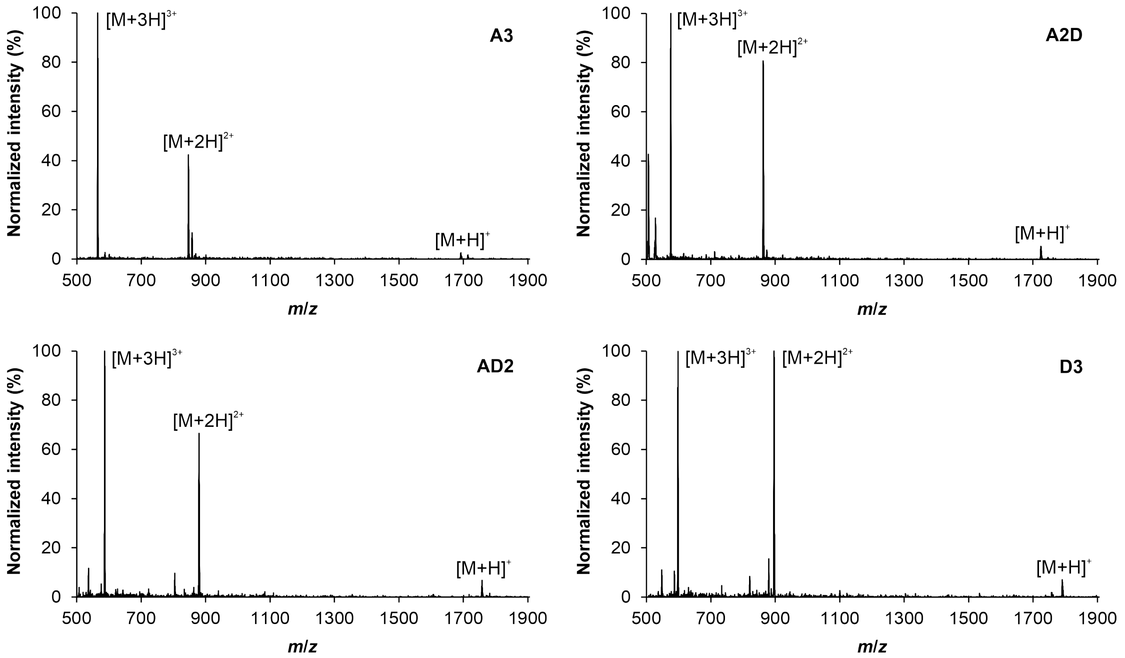

Figure S16. Mass spectra of **A3**, **A2D**, **AD2** and **D3**.

Table S4. Calculated and found mass (*m*/*z*) of the ions of **zD'XXXp**.

Oligomers	Formula	Calcd. *m*/*z*	Found *m*/*z*
	$[M+H]^+$	1691.13	1691.94
**A3**	$[M+2H]^{2+}$	846.07	846.40
	$[M+3H]^{3+}$	564.38	564.70
	$[M+H]^+$	1724.11	1724.39
**A2D**	$[M+2H]^{2+}$	862.55	862.84
	$[M+3H]^{3+}$	575.37	575.70
	$[M+H]^+$	1757.08	1758.33
**AD2**	$[M+2H]^{2+}$	879.04	879.41
	$[M+3H]^{3+}$	586.36	586.70
	$[M+H]^+$	1790.05	1790.34
**D3**	$[M+2H]^{2+}$	895.53	895.91
	$[M+3H]^{3+}$	597.35	597.70

Amount of isolated library of oligomers: The library of oligomers **zD'XXXp** (1 mg) was dissolved in DMSO-d_6 with 1,3,5-trimethoxybenzene as the internal standard (I.S.). A ^{1}H NMR spectrum of the mixture was acquired at 373 K (Figure S17 and Figure S18). Based on the integral value of the selected proton signal (1, Figure S13), which was present in all oligomers of the library, and those of the internal standard, we determined that 12.5 μmol of library of oligomers were isolated after purification.

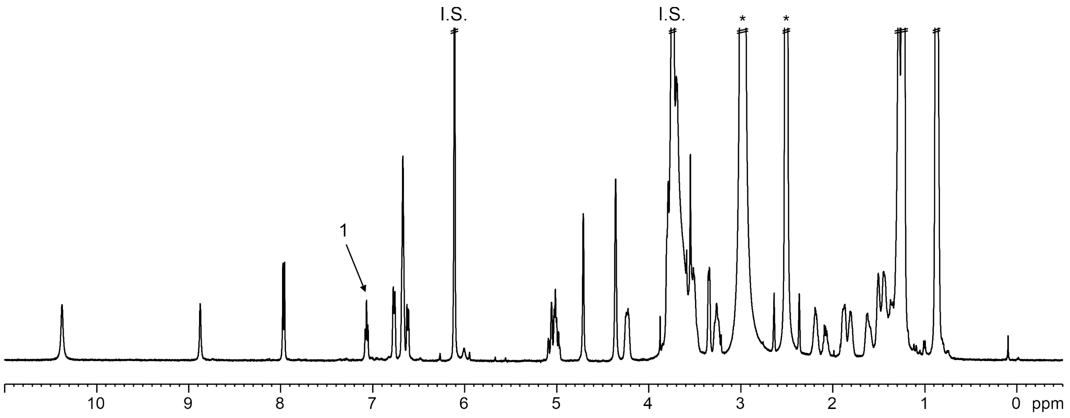

Figure S17. ^{1}H NMR (500 MHz, DMSO-d_6, 373 K) spectrum of the library of oligomers **zD'XXXp** after purification. See Figure S13 for proton assignment. I.S. = 1,3,5-trimethoxybenzene. *Residual solvent peaks.

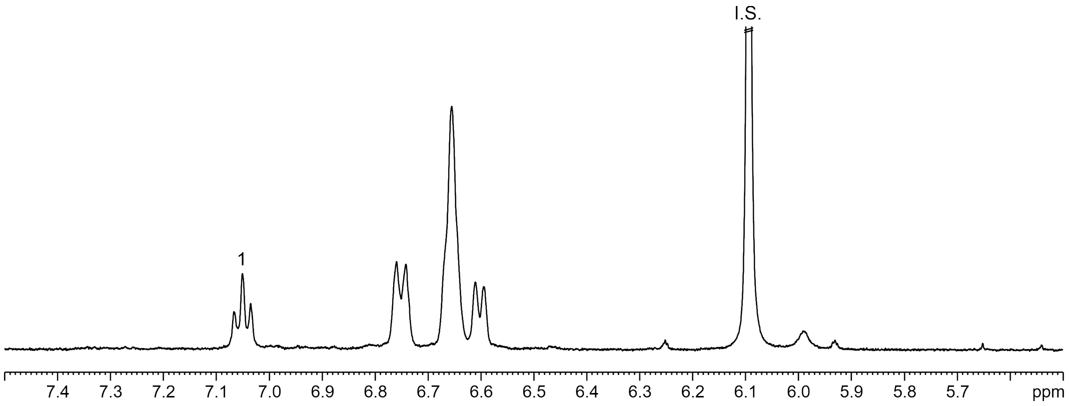

Figure S18. Selected region of the ^{1}H NMR (500 MHz, DMSO-d_6, 373 K) spectrum of the library of oligomers **zD'XXXp** after purification. See Figure S13 for proton assignment. I.S. = 1,3,5-trimethoxybenzene.

6. Covalent capture of oligomers

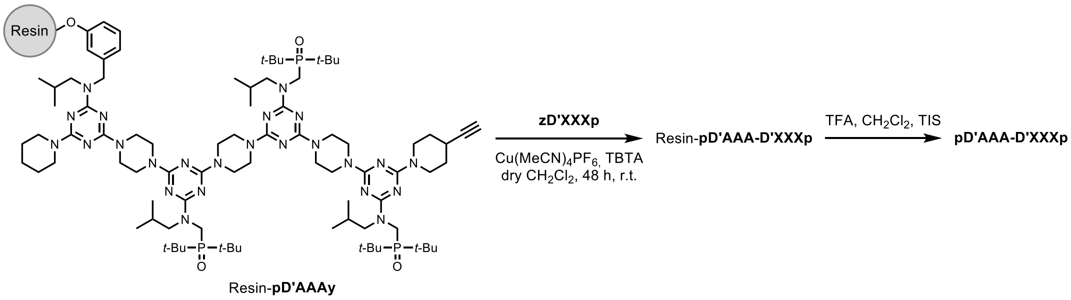

Scheme S5. Covalent capture of melamine oligomers with the alkyne-oligomer attached to the resin.

General procedure: Resin-**pD'AAAy** (*ca.* 8 mg, *ca.* 0.5 µmol) was suspended in dry CH_2Cl_2 (3.8 mL) in a 5 mL glass vial. The reacting azide compound/s (1 µmol for each melamine oligomer) was dissolved in dry CH_2Cl_2 (1 mL) and added to the resin suspension. Finally, $Cu(MeCN)_4PF_6$ (0.1 mL from a 10 mM CH_2Cl_2 stock solution, 1 µmol) and TBTA (0.1 mL from a 10 mM CH_2Cl_2 stock solution, 1 µmol) were added. The glass vial was sealed, and the reaction was shaken at r.t. for 48 h. After that, the crude was filtered using a 2 mL plastic syringe equipped with a frit, and the resin was washed with CH_2Cl_2 (5 × 1 mL), DMF (20 × 1 mL) and then CH_2Cl_2 (10 × 1 mL). The oligomers were cleaved from the resin with 90:5:5 TFA/CH_2Cl_2/TIS (1 mL) at r.t. for 2 h. The crude was filtered, and the resin was washed with CH_2Cl_2 (5 × 1 mL). The crude was concentrated with a nitrogen flow, redissolved in 2:1 DMSO/THF (1.5 mL) and analyzed by UPLC-MS.

6.1 Reaction with the azide-oligomer containing three 4-nitrophenol units

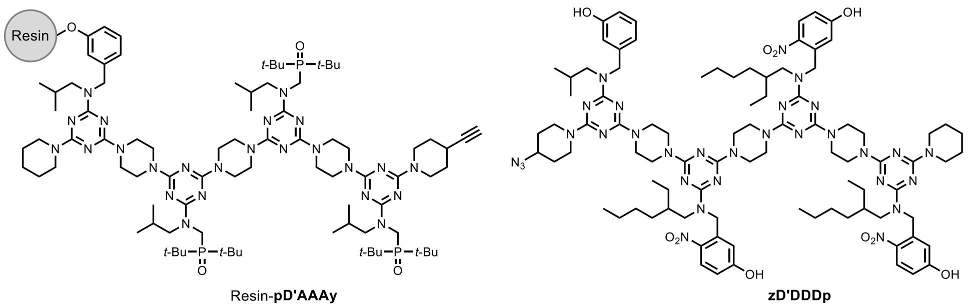

Figure S19. Line-drawing structures of Resin-**pD'AAAy** and **zD'DDDp** used in the covalent capture experiment.

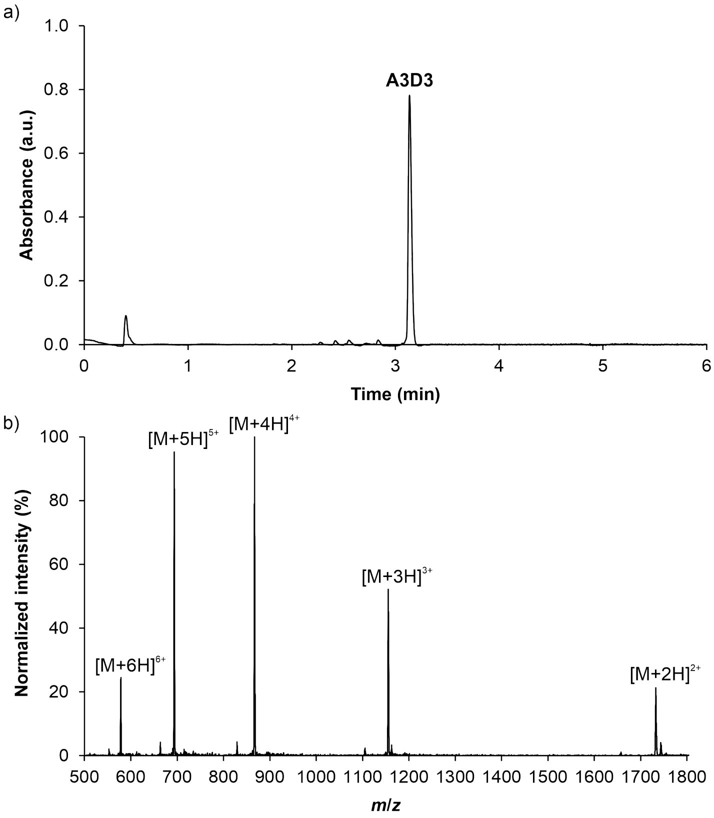

Figure S20. a) UPLC chromatogram of the crude reaction mixture between Resin-**pD'AAAy** and **zD'DDDp** after filtration, washing and cleavage. Method (water/THF, containing 0.1% formic acid): 70:30 → 0:100 in 4 min and 0:100 for 2 min; flow rate = 0.4 mL·min^{-1}. b) Mass spectrum of **A3D3**.

Table S5. Calculated and found mass (*m*/*z*) of the ions of **A3D3**.

Formula	Calcd. *m*/*z*	Found *m*/*z*
$[M+2H]^{2+}$	1732.09	1732.76
$[M+3H]^{3+}$	1155.06	1155.62
$[M+4H]^{4+}$	866.55	867.03
$[M+5H]^{5+}$	693.44	693.77
$[M+6H]^{6+}$	578.04	578.51

6.2 Reaction with the azide-oligomer containing three phosphine oxide units

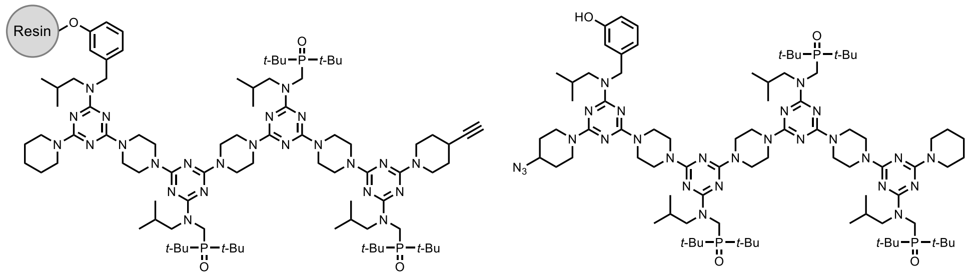

Resin-**pD'AAAy** **zD'AAAp**

Figure S21. Line-drawing structures of Resin-**pD’AAAy** and **zD’AAAp** used in the covalent capture experiment.

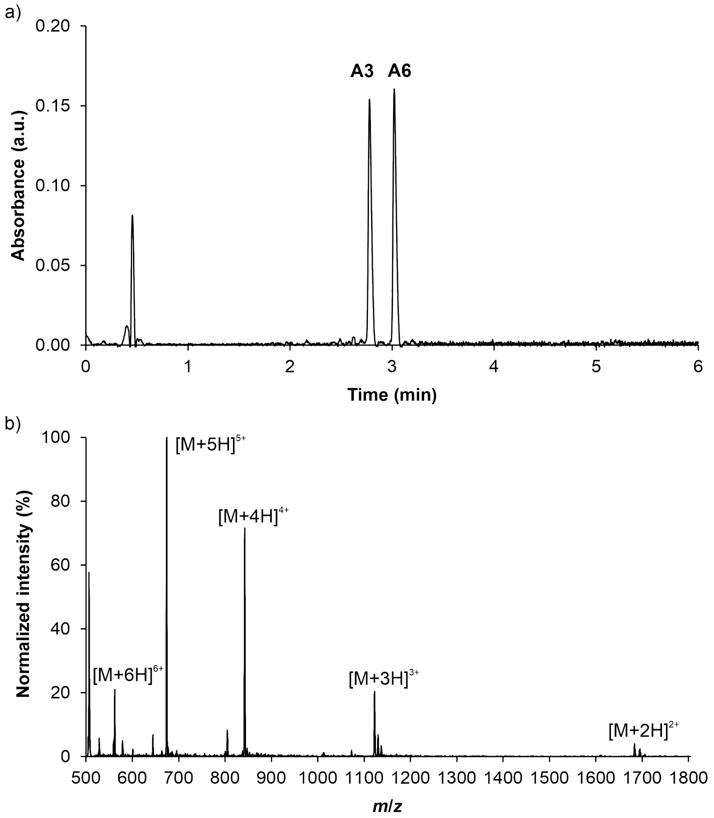

Figure S22. a) UPLC chromatogram of the crude reaction mixture between Resin-**pD’AAAy** and **zD’AAAp** after filtration, washing and cleavage. Method (water/THF, containing 0.1% formic acid): 70:30 → 0:100 in 4 min and 0:100 for 2 min; flow rate = 0.4 mL·min^{-1}. b) Mass spectrum of **A6**.

Table S6. Calculated and found mass (*m*/*z*) of the ions of **A6**.

Formula	Calcd. *m*/*z*	Found *m*/*z*
$[M+2H]^{2+}$	1682.64	1683.82
$[M+3H]^{3+}$	1122.09	1122.74
$[M+4H]^{4+}$	841.82	842.32
$[M+5H]^{5+}$	673.66	674.14
$[M+6H]^{6+}$	561.55	562.13

6.3 Reaction with a mixture of two azide-oligomers

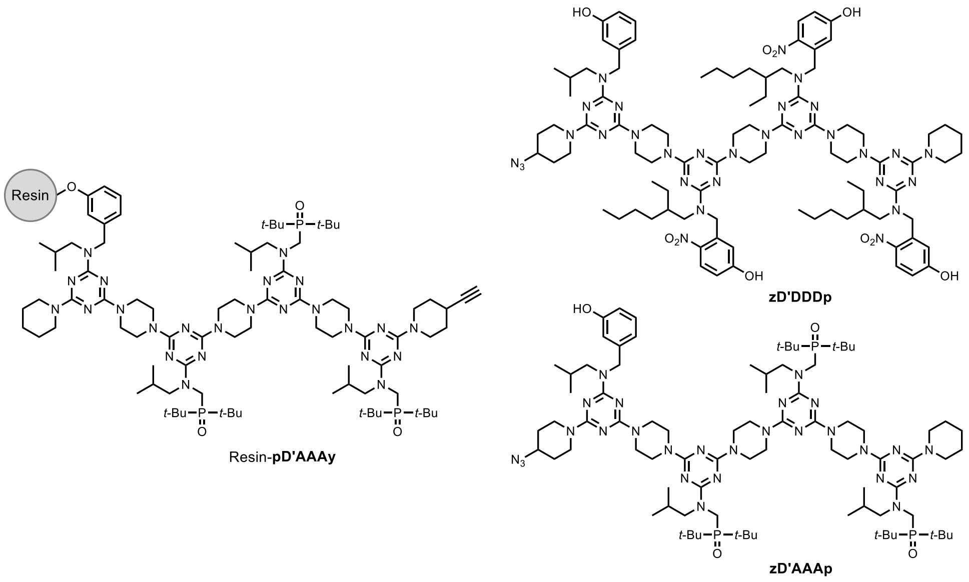

Figure S23. Line-drawing structures of Resin-**pD’AAAy**, **zD’DDDp** and **zD’AAAp** used in the competition experiment.

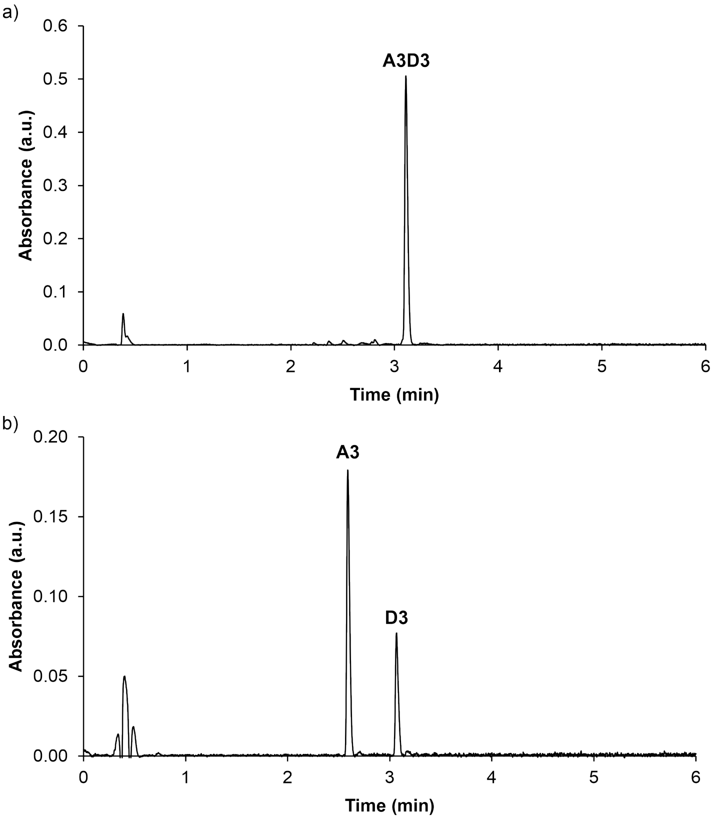

Figure S24. UPLC chromatograms: a) crude reaction mixture between Resin-**pD’AAAy**, **zD’DDDp** and **zD’AAAp** after filtration, washing and cleavage and b) solution mixture obtained after filtration and washing. Method (water/THF, containing 0.1% formic acid): 70:30 → 0:100 in 4 min and 0:100 for 2 min; flow rate = 0.4 mL·min^{-1}.

6.4 Reactions with a randomized library of azide-oligomers

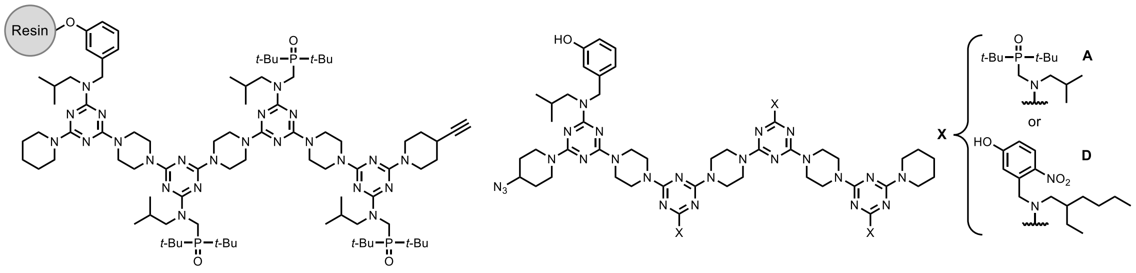

Figure S25. Line-drawing structures of Resin-**pD'AAAy** and **zD'XXXp** used in the covalent capture experiment. Note that the randomized library was composed of 8 oligomers: **A3**, 3 × **A2D**, 3 × **AD2** and **D3**.

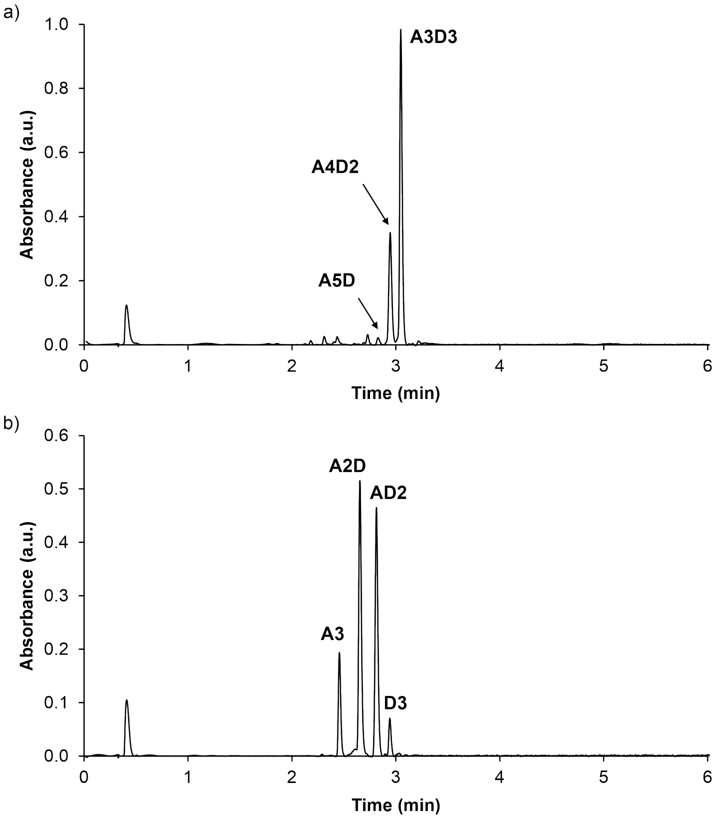

Figure S26. UPLC chromatograms: a) crude reaction mixture between Resin-**pD'AAAy** and **zD'XXXp** after filtration, washing and cleavage and b) solution mixture obtained after filtration and washing. Method (water/THF, containing 0.1% formic acid): 70:30 → 0:100 in 4 min and 0:100 for 2 min; flow rate = 0.4 mL·min^{-1}.

Table S7. Composition (%) of the crude reaction mixture after filtration, washing and cleavage.

Oligomers	Composition (%)
**A3D3**	*ca.* 75
**A4D2**	*ca.* 25
**A5D**	trace
**A6**	not observed

Modified procedure: Resin-**pD'AAAy** (*ca.* 4 mg, *ca.* 0.25 µmol) was suspended in dry CH_2Cl_2 (23 mL) in a 25 mL glass vial. The library of oligomers **zD'XXXp** (4 µmol) was dissolved in dry CH_2Cl_2 (1 mL) and added to the resin suspension. Finally, $Cu(MeCN)_4PF_6$ (0.5 mL from a 10 mM CH_2Cl_2 stock solution, 5 µmol) and TBTA (0.5 mL from a 10 mM CH_2Cl_2 stock solution, 5 µmol) were added. The next steps were identical to those described in the General procedure.

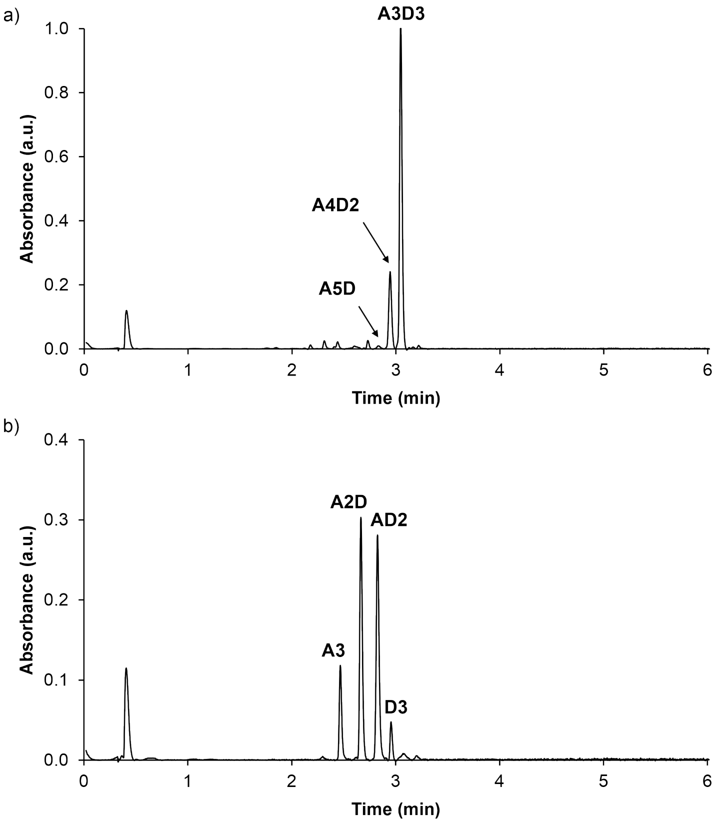

Figure S27. UPLC chromatograms: a) crude reaction mixture between Resin-**pD'AAAy** and **zD'XXXp** after filtration, washing and cleavage (modified procedure) and b) solution mixture obtained after filtration and washing. Method (water/THF, containing 0.1% formic acid): 70:30 → 0:100 in 4 min and 0:100 for 2 min; flow rate = 0.4 mL·min^{-1}.

Table S8. Composition (%) of the crude reaction mixture after filtration, washing and cleavage.

Oligomers	Composition (%)
**A3D3**	*ca.* 80
**A4D2**	*ca.* 20
**A5D**	trace
**A6**	not observed

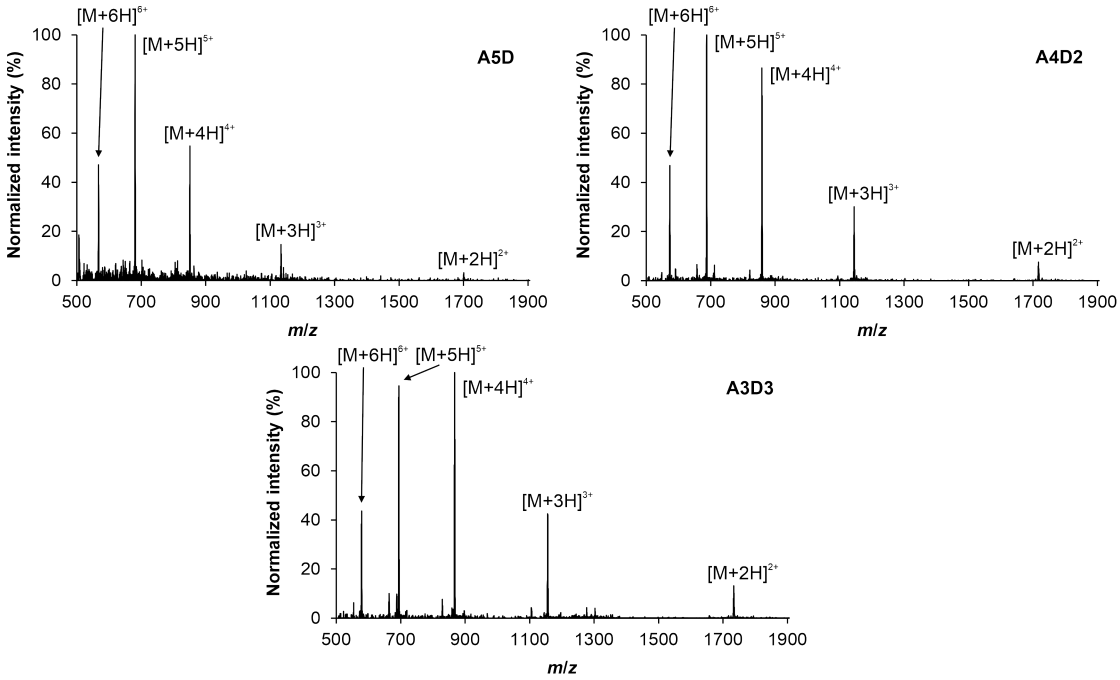

Figure S28. Mass spectra of **A5D**, **A4D2** and **A3D3**.

Table S9. Calculated and found mass (*m*/*z*) of the ions of **A5D**, **A4D2** and **A3D3**.

Oligomers	Formula	Calcd. *m/z*	Found *m/z*
	$[M+2H]^{2+}$	1699.12	1700.88
	$[M+3H]^{3+}$	1133.08	1133.80
**A5D**	$[M+4H]^{4+}$	850.06	850.90
	$[M+5H]^{5+}$	680.25	680.58
	$[M+6H]^{6+}$	567.05	567.26
	$[M+2H]^{2+}$	1715.60	1716.51
	$[M+3H]^{3+}$	1144.07	1144.80
**A4D2**	$[M+4H]^{4+}$	858.30	858.90
	$[M+5H]^{5+}$	686.85	687.20
	$[M+6H]^{6+}$	572.54	573.13
	$[M+2H]^{2+}$	1732.09	1733.45
	$[M+3H]^{3+}$	1155.06	1155.93
**A3D3**	$[M+4H]^{4+}$	866.55	867.22
	$[M+5H]^{5+}$	693.44	693.02
	$[M+6H]^{6+}$	578.04	578.63

7. References

[1] G. R. Fulmer, A. J. M. Miller, N. H. Sherden, H. E. Gottlieb, A. Nudelman, B. M. Stoltz, J. E. Bercaw and K. I. Goldberg, *Organometallics*, 2010, **29**, 2176-2179.

[2] D. Imperio, G. B. Giovenzana, G.-I. Law, D. Parker and J. W. Walton, *Dalton Trans*., 2010, **39**, 9897-9903.

[3] G. Iadevaia, A. E. Stross, A. Neumann and C. A. Hunter, *Chem. Sci*., 2016, **7**, 1760-1767.

[4] H. Sakai, H. Inoue, T. Toba, K. Murata, N. Narii, Y. Shimmyo, Y. Igawa, T. Matsumoto and N. Takemoto, *Bioorg. Med. Chem. Lett*., 2019, **29**, 2332-2337.

[5] P. Troselj, P. Bolgar, P. Ballester and C. A. Hunter, *J. Am. Chem. Soc*., 2021, **143**, 8669-8678.

[6] M. Dhiman, R. Cabot and C. A. Hunter, *Chem. Sci*., 2024, **15**, 5957-5963.

[7] M. Dhiman, R. Cons, O. N. Evans, J. T. Smith, C. J. Anderson, R. Cabot, D. O. Soloviev and C. A. Hunter, *J. Am. Chem. Soc*., 2024, **146**, 9326-9334.

[8] F. Balduzzi, V. Munasinghe, O. N. Evans, A. Lorusso Notaro Francesco, C. J. Anderson, S. Nigrelli, L. Escobar, R. Cabot, J. T. Smith, C. A. Hunter, J. Am. Chem. Soc., 2024, DOI: 10.1021/jacs.4c13452.
